# Transcriptomic and functional pathways analysis of ascorbate-induced cytotoxicity and resistance of Burkitt lymphoma

**DOI:** 10.18632/oncotarget.11740

**Published:** 2016-08-31

**Authors:** Zenglin Pei, Xuan Zhang, Chunxia Ji, Song-Mei Liu, Jin Wang

**Affiliations:** ^1^ Scientific Research Center, Shanghai Public Health Clinical Center, Jinshan District, Shanghai 201508, China; ^2^ Center for Gene Diagnosis, Zhongnan Hospital of Wuhan University, Wuhan, Hubei 430071, China

**Keywords:** drug resistance, transcriptomic profiles, pathway analysis, ascorbate, Burkitt lymphoma cells

## Abstract

Ascorbate is a pro-oxidant that generates hydrogen peroxide–dependent cytotoxity in cancer cells without adversely affecting normal cells. To determine the mechanistic basis for this phenotype, we selected Burkitt lymphoma cells resistant to ascorbate (JLPR cells) and their ascorbate-sensitive parental cells (JLPS cells). Compared with JLPS cells, the increased glucose uptake in JLPR cells (with upregulated glucose transporters, increased antioxidant enzyme activity, and altered cell cycling) conferred ascorbate–induced cytotoxicity and resistance. Transcriptomic profiles and function pathway analysis identified differentially expressed gene signatures for JLPR cells and JLPS cells, which differential expression levels of five genes (*ATF5, CD79B, MHC, Myosin*, and *SAP18*) in ascorbate-resistant cells were related to phosphoinositide 3 kinase, *cdc42*, DNA methylation and transcriptional repression, polyamine regulation, and integrin-linked kinase signaling pathways. These results suggested that coordinated changes occurred in JLPR cells to enable their survival when exposed to the cytotoxic pro-oxidant stress elicited by pharmacologic ascorbate treatment.

## INTRODUCTION

Ascorbate (Vitamin C) is a nutrient essential to the biosynthesis of collagen and L-carnitine and the conversion of dopamine to norepinephrine [1]. Most animals are able to synthesize large quantities of ascorbate, but humans lost this ability due to mutations of the gulonolactone oxidase gene [2]. It is well known that an acute lack of ascorbate leads to scurvy and causes death [3] and ascorbate is an attractive marker of fruit and vegetable intake because these foods are the primary sources of dietary vitamin C [4], which ascorbate functions as a potent reducing agent that efficiently quenches potentially damaging free radicals [5]. It is very interesting that Cameron suggested that high doses of ascorbate could provide a clinical benefit for cancer patients [6, 7]. *In vitro* studies have shown that pharmacologic ascorbate is effective in a large panel of tumor cell lines [8, 9] and that increasing tumor cells' generation of hydrogen peroxide (H_2_O_2_) might be used to induce ascorbate-specific cytotoxicity [10]. Pharmacologic ascorbate has been found to mediate the mitochondrial release of cytochrome C, which leads to H_2_O_2_-mediated activation of the caspase cascade and apoptotic process and thence to a significant decrease in the growth rate of some solid tumors [11–14]. Herst investigated that 5 mM ascorbate in combination with radiation killed more glioblastoma multiforme primary cells by increasing oxidative DNA damage than either treatment alone [15]. However, the effect of high doses of intravenous ascorbate in the treatment of cancer has been controversial although there is growing evidence that intravenous high-dose ascorbate has been found to improve the health-related quality of life of terminal cancer patients [16,17].

On another hand, some normal human cells (lymphocytes, monocytes, fibroblasts and normal breast cells) and some types of human cancer cells (breast cancer cells and glioblastoma multiforme cells) are more resistant to ascorbate than others [8, 9]. Moreover, ascorbate penetrating the tissue at a slightly lower rate than mannitol and travelling via the paracellular route were also found [18]. Compared with human renal carcinoma TC-1 cells, higher intracellular glutathione concentration was detected in glioblastoma multiform tumor T98G cells, which were more resistant to ascorbate than TC-1 cells [9]. Sinnberg also found that ascorbate resistance was associated with the expression of HIF1α and oxygen pressure [19]. Therefore, the use of ascorbate as an anticancer agent and ascorbate-induced resistance warrants further study. The mechanisms of drug resistance in cancer cells have been thoroughly studied at the gene transcription levels by cDNA microarrays, which is a high-throughput system developed to monitor the expression of many genes in a single experiment and to identify genes differentially expressed in multidrug-resistant cancer cells and those of their parental cells [20–22]. In this study, we used transcriptomic profiling, quantitative real-time polymerase chain reaction (qRT-PCR), and antioxidant enzyme activity assays to clarify the molecular mechanism of ascorbate-induced resistance in Burkitt lymphoma cells.

## RESULTS

### Cytotoxic response of JLPS and JLPR cells to ascorbate or H_2_O_2_

**Figure 1:**
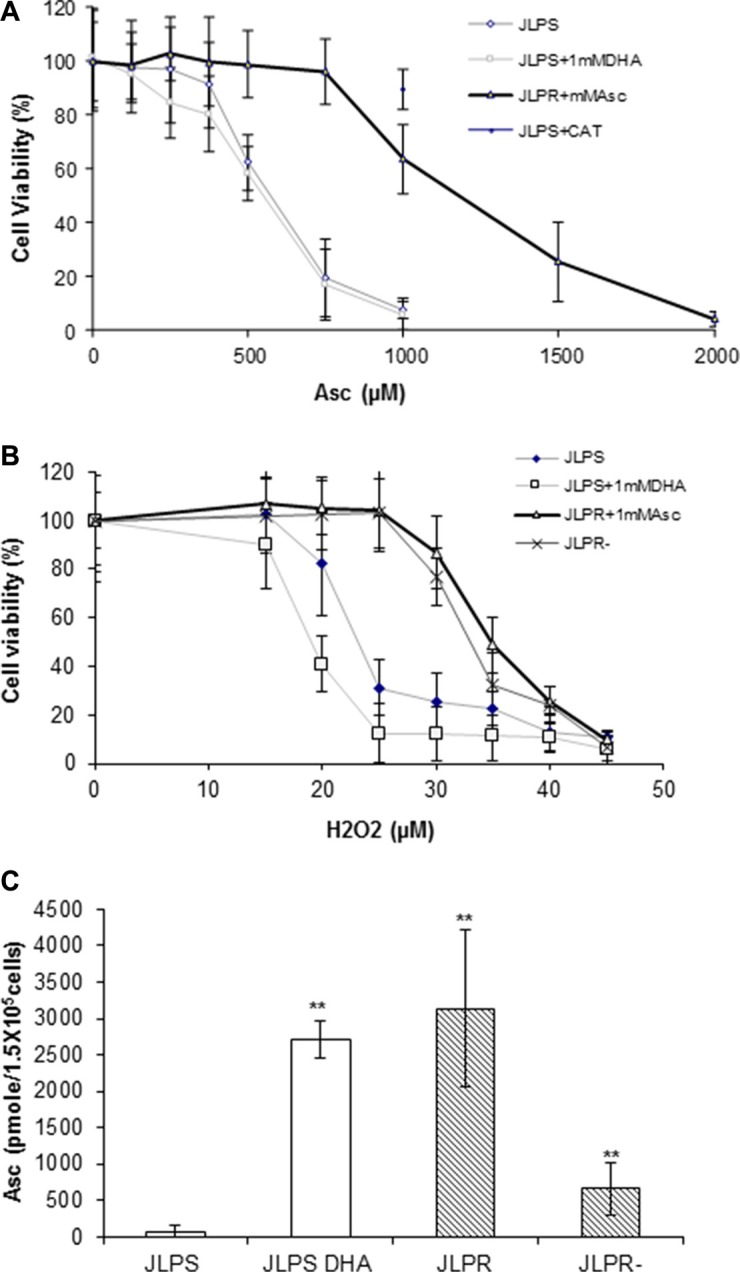
Cytotoxicity analysis of ascorbate and H_2_O_2_ with the measurement of intracellular ascorbate in JLPR cells and JLPS cells (**A**) Viability rates of JLPS cells, JLPS cells pretreated with DHA (JLPS+DHA), JLPR cells, and JLPS cells pretreated with CAT following treatment with increasing concentrations of ascorbate. (**B**) Viability rates of JLPS cells, JLPS+DHA, JLPR cells, and JLPR cells that had not been maintained in ascorbate (JLPR−) following treatment with increasing concentrations of H_2_O_2_. (**C**) Posttreatment concentrations of ascorbate in JLPS cells, JLPS+DHA cells, JLPR cells, and JLPR− cells.

Using the MTT assay, we found that JLPR cells were highly resistant to ascorbate and H_2_O_2_ at IC_50_ values of 1250 μM and 32 μM, respectively (Figure 1A, Figure 1B, and Table 1). High-performance liquid chromatography analysis revealed that JLPR cells had a larger amount of ascorbate than JLPS cells did (Figure 1C). JLPS cells incubated with 1 mM docosahexanoic acid (DHA) for 1 h had a similar amount of ascorbate as JLPR cells did (Figure 1C). Because cellular ascorbate cannot induce the death of JLP cells, there was almost no difference in cell viability rates between JLPS cells treated with ascorbate and JLPS cells treated with ascorbate and DHA (Figure 1A). JLPS cells pretreated with CAT acquired some resistance to ascorbate (Figure 1A). After 48 h, JLPR cells that had not been maintained in 1 mM ascorbate lost little H_2_O_2_ resistance (Figure 1B). These results suggested that ascorbate efflux has an antioxidation function that protects cells from H_2_O_2_-induced cytotoxicity and that ascorbate resistance is associated with progressive accumulation of reactive oxygen species.

**Table 1:** Cytotoxicity studies of JLP cells by MTT assay (IC_50_)

Drug	JLP S	JLPR	Resistance index^a^
	IC_50_	IC_50_	
Ascorbate	375 μM	1250 μM	3.33
H_2_O_2_	16 μM	32 μM	2.00

Resistance index

a the ratio of the IC_50_ for JLPR treatment with ascorbate/ H_2_O_2_ to the IC_50_ for JLPS treatment with ascorbate.

### Increased glucose uptake in JLPR cells with activation of glucose transport

**Figure 2:**
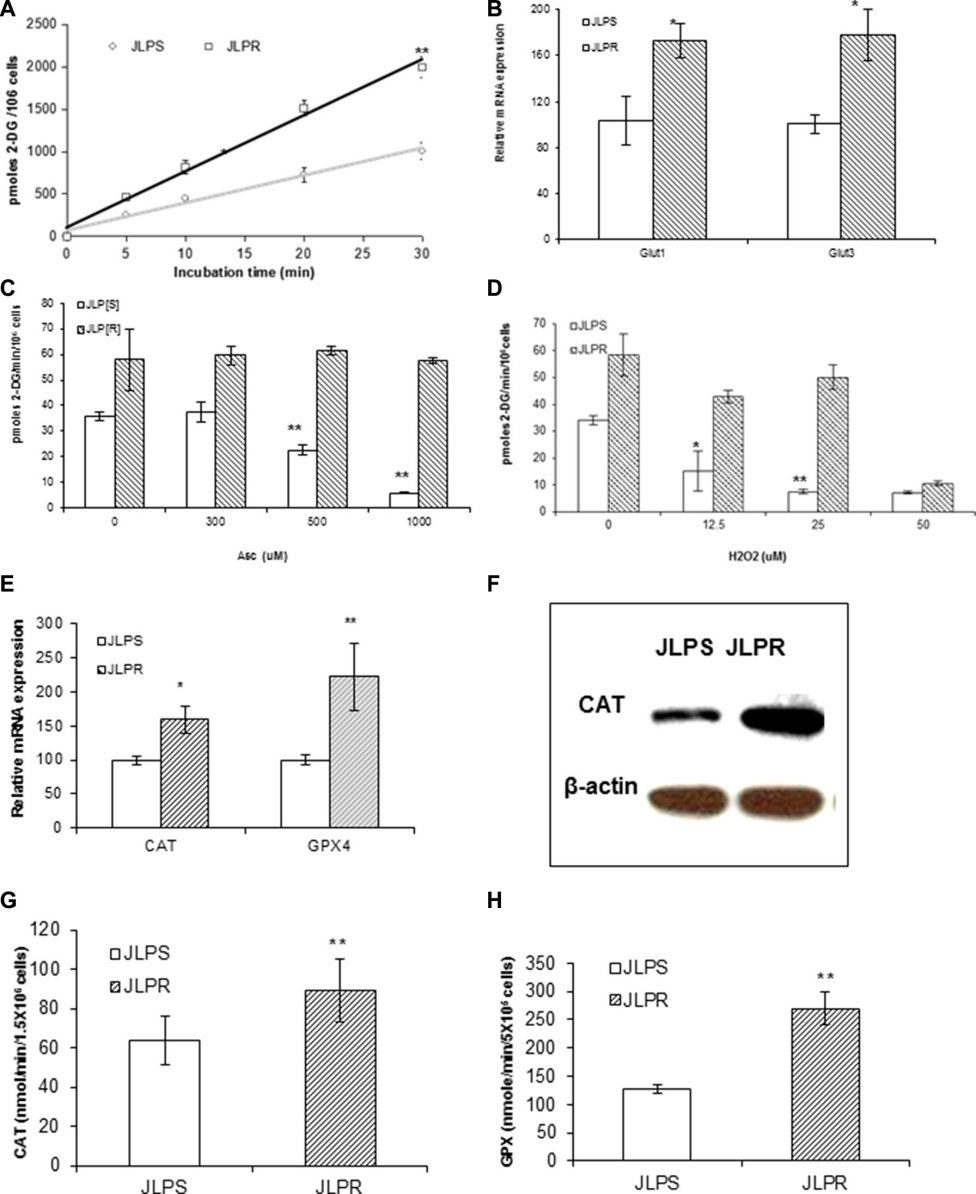
Metabolomic assay and qRT -PCR analysis in JLPR cells and JLPS cells (**A**) 2-DG uptake at different times. (**B**) Real-time PCR analysis of Glut1 and Glut3 mRNA. (**C**) 2-DG uptake in cells treated with increasing concentrations of ascorbate. (**D**) 2-DG uptake in cells treated with increasing concentrations of H_2_O_2_. Antioxidant enzyme analysis in JLPS and JLPR cells. (**E**) qRT-PCR analysis of CAT and GPX4 gene expression. (**F**) Western blot analysis of CAT protein expression levels. (**G**) CAT activity. (H) GPX4 activity.

The amount of 2-DG uptake at different times in JLPR cells was higher than that in JLPS cells (Figure 2A). To determine whether the changes in glucose transport occurred at the level of gene transcription, we analyzed the expressions of *Glut1* and *Glut3*. Real-time PCR revealed that the expression levels of *Glut1* and *Glut3* in JLPR cells were 1.72- and 1.77-fold higher, respectively, than those in JLPS cells (Figure 2B). We found that following treatment with ascorbate and H_2_O_2_, the amount of glucose uptake in JLPS cells was usually significantly lower than that in JLPR cells (*P* < 0.05). The amount of 2-DG uptake was decreased by 78.57% (from 35 to 7.5 pmoles 2-DG/min/10^6^ cells) in JLPS cells treated with 1000 μM ascorbate or 25 μM H_2_O_2_ (Figure 2C and 2D). Our results demonstrated that ascorbate treated with cancer cells by the role of H_2_O_2_ in mediating glucose uptake activation in JLPS cells. On the other hand, the resistant JLPR cells maintained high glucose uptake (about 55 pmoles 2-DG/min/10^6^ cells) despite being treated in 1 mM ascorbate (Figure 2C) or 25 μm H_2_O_2_ (Figure 2D). These results suggested that ascorbate resistance actively restores glucose uptake against H_2_O_2_-induced apoptosis and necrosis.

### Increased levels of antioxidant enzyme (*CAT* and *GPX*) mRNA, protein, and activity in JLPR cells

qRT-PCR was used to assess the levels of antioxidant enzyme mRNA expression. The expression levels of *CAT* and *GPX4* mRNA in JLPR cells were higher than those in the JLPS cells (Figure 2E). Immunoblots analysis revealed *CAT* protein levels to be higher in JLPR cells than JLPS cells (Figure 2F). The *CAT* and *GPX* activities of JLPR cells (*CAT*: 59.6 nmoles/min/10^6^ cells; *GPX*: 538.6 nmoles/min/10^6^ cells) were 1.5 and 2 times as high as those of JLPS cells (*CAT*: 42.3 nmoles/min/10^6^ cells; *GPX*: 253.6 nmoles/min/10^6^ cells), respectively (Figure 2G and Figure 2H). These results were consistent with the findings regarding mRNA and protein levels. These findings indicated that the activities of the *CAT* and *GPX* enzymes are involved in protecting cells against oxidative stress, which suggests that overexpression of *CAT* and *GPX* confers resistance to ascorbate.

### Ascorbate and H_2_O_2_ trigger similar models of cell cycling, but JLPR cells do not arrest in the G_2_/M phase induced by ascorbate or H_2_O_2_

**Figure 3:**
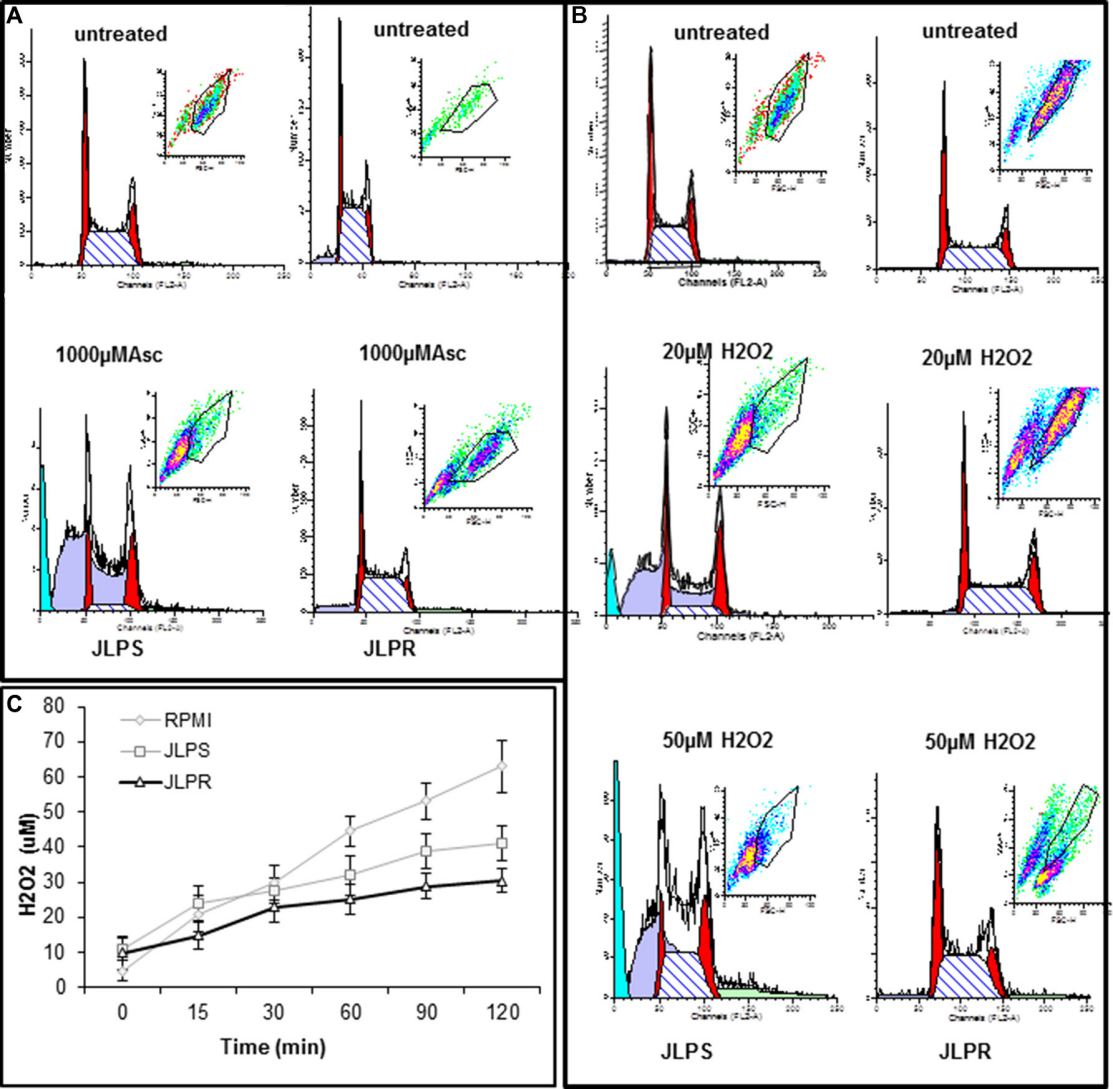
Cellular DNA content in JLPS and JLPR cells following treatment with ascorbate and H_2_O_2_ (**A**) Cells following treatment with 0 μM or 1000 μM ascorbate. (**B**) Cells following treatment with 0 μM, 20 μM or 50 μM H_2_O_2_. (**C**) H_2_O_2_ production in RPMI 1640 medium alone, containing JLPS cells, or containing JLPR cells. Ascorbate (1 mM) was added and medium was incubated at room temperature for up to 120 min. Data are means ± standard deviations of three independent experiments.

Propidium iodide staining and flow cytometry were used to assess the DNA content of untreated and ascorbate- or H_2_O_2_-treated JLPS cells and JLPR cells. Following treatment with 1 mM ascorbate or 20 μM H_2_O_2_, JLPS cells were arrested predominantly in the G_2_/M phase; a few cells arrested in the G1 phase (Figure 3A and 3B). These results demonstrated that JLPS cells arrest at the G2/M phase, whereas JLPR cells do not, which implied that JLPR cells are more effective in removing H_2_O_2_ from the treatment medium. Interestingly, the cell cycling of JLPS cells treated with ascorbate was basically identical to that of JLPS cells treated with H_2_O_2_. These findings indicated that JLPS cells, but not JLPR cells, arrest in the G_2_/M phase in response to ascorbate or H_2_O_2_ and that ascorbate and H_2_O_2_ trigger similar models of cell killing in JLPS cells.

### Ascorbate generates extracellular H_2_O_2_, which induces cancer cells' cytotoxic pro-oxidant stress

An O_2_ electrode assay was used to measure the amount of H_2_O_2_ in complete culture medium with or without JLPS or JLPR cells after dissolution of ascorbate. The assay revealed that the amount of H_2_O_2_ in the presence of cells, especially JLPR cells, was less than that in medium alone (Figure 3C). The amount of H_2_O_2_ plateaued in JLPS cells treated with ascorbate for 1 h or more. These findings indicated that an ascorbate oxidation process produces extracellular H_2_O_2_. In the presence of JLPS cells in RPMI medium, 1 mM ascorbate generated 38 μM H_2_O_2_, arrested JLPS cells in the G_2_/M phase, and induced 90% cytotoxicity. In the presence of JLPR cells, 1 mM ascorbate generated 30 μM H_2_O_2_ but did not arrest cells in the G_2_/M phase or cause cell death (Figure 3A).

### Transcriptomic profiles in JLP cells

To identify possible mechanisms of ascorbate resistance, we performed gene microarray profiling of JLPS and JLPR cells. A genome-wide analysis of the gene transcripts expressed in both cell lines was performed using agilent array. Hybridization of the microarray data revealed that 1.82% of the cDNAs exhibited more than a 1.5-fold change in expression level (*P* ≤ 0.05). The genes and genetic networks involved in ascorbic resistance are described in Tables 2 and 3, respectively. The microarray experiments also revealed that the following genes and genetic networks play roles in acquired ascorbate resistance: the double-strand break repair genes *TOP2B* and *XRCC5*; ferritin genes *FTL*, *FTH1*, and *FTHL2*; antioxidation gene *GPX4*; major histocompatibility complex (*MHC*) class I genes human leukocyte antigen *HLA-A, HLA-G*, and *HLA-H*; and asparagine synthetase; the downregulation of the histone genes *HIST1H2AE, HIST1H2BK, H2BFS*, and *HIST1H4B*; histone deacetylase (*HDAC*) complex subunit SAP18; heat shock protein (*HSP*) genes *HSPH1* and *HSPE1*; *FLAP*; the inhibition of tumor necrosis and apoptosis signaling by inhibiting the expression of high-mobility group protein box 1 (*HMGB1*) and v-Myc myelocytomatosis viral oncogene homolog (*MYC*); and the increased expression of activating transcription factor 5 (*ATF5*) at the gene transcription level (Supplementary Table S1).

**Table 2:** Genetic networks associated with ascorbate-resistance in JLPR cells

Associated network functions	Score	Focus molecules	Molecules in network
Cell-to-cell signaling and interaction, hematological system development and function, immune cell trafficking	47	18	Akt, ASNS, **ATF5**, BCR, c-Myc/N-Myc, **C5orf13**, CD3, **CD79B**, Ck2, E2f, ERK1/2, **FTL, HIST1H2AB/HIST1H2AE, HIST1H2BJ/HIST1H2BK,** HISTONE, **HLA-A, HMGB1, HSPH1,** IFN Beta, **IGLL1/IGLL5,** Immunoglobulin, Insulin, Interferon alpha, KIAA0020, MHC CLASS I (family), **MYC**, NFkB (complex), PI3K (complex), **RPS7, SAP18, SSX1, TCL1A, TKT, TOP2B**, Ubiquitin
Cell death, cell-to-cell signaling and interaction, hematological system development and function	22	10	**ALOX5AP, ATF5**, BATF, beta-estradiol, CD6, DDIT3, FGF2, Gm4617 /Ptma, GRB2, HIF1A, HLA Class I, HPS1, **HPS4**, IDH2, IFI30, IFNG, KRT6A, LGMN, **MAMLD1**, mir-23, miR-199a-5p, MLANA, **MYL6**, Myosin Light Chain Kinase, NCAN, NCR3, **POMP**, RAC1, SLK, **SSX2, SSX4/SSX4B,** Tgtp1, **TMEM45A**, TNF, **ZFP57**

**Table 3:** Top 5 canonical pathways involving genes differentially expressed in JLPR cells

Signaling pathway	*P* value	Ratio	Molecules
PI3K signaling in B lymphocytes	1.95 E-02	2:147 (0.014)	CD79B, ATF5
Cdc42 signaling	2.77 E-02	2:180 (0.011)	MLC, MHC
DNA methylation and transcriptional repression signaling	3.14 E-02	1:23 (0.043)	SAP18
Polyamine regulation in colon cancer	3.45 E-02	1:29 (0.034)	C-Myc
ILK signaling	3.49 E-02	2:193 (0.01)	C-Myc, Myosin

### Functional networks and pathways of ascorbate-induced drug resistance

The genetic networks and cellular pathways deregulated in JLPR cells were identified using the IPA software program. Expression microarray profiling studies revealed that 45 genes were deregulated in JLPR cells (Supplementary Table S1). A comprehensive network and pathway analysis of the deregulated genes revealed that these genes were associated with two network functions and five canonical pathways relevant to the development of ascorbate resistance in cancer cells. In each of the two genetic networks, the differently expressed genes constituted about half of the molecules involved in network-associated cellular functions and included genes related to cell-to-cell signaling and interaction, cell death, hematological system development and function, and immune cell trafficking (Table 2). These genes are expected to be affected during ascorbate resistance development in Burkitt lymphoma cells. The differentially expressed genes belong to five canonical signaling pathways that are frequently deregulated in ascorbate-resistant cells (Table 3).

### qRT-PCR and immunobloting analyses validation of differently expressed oxidative stress genes in JLP cells

**Figure 4:**
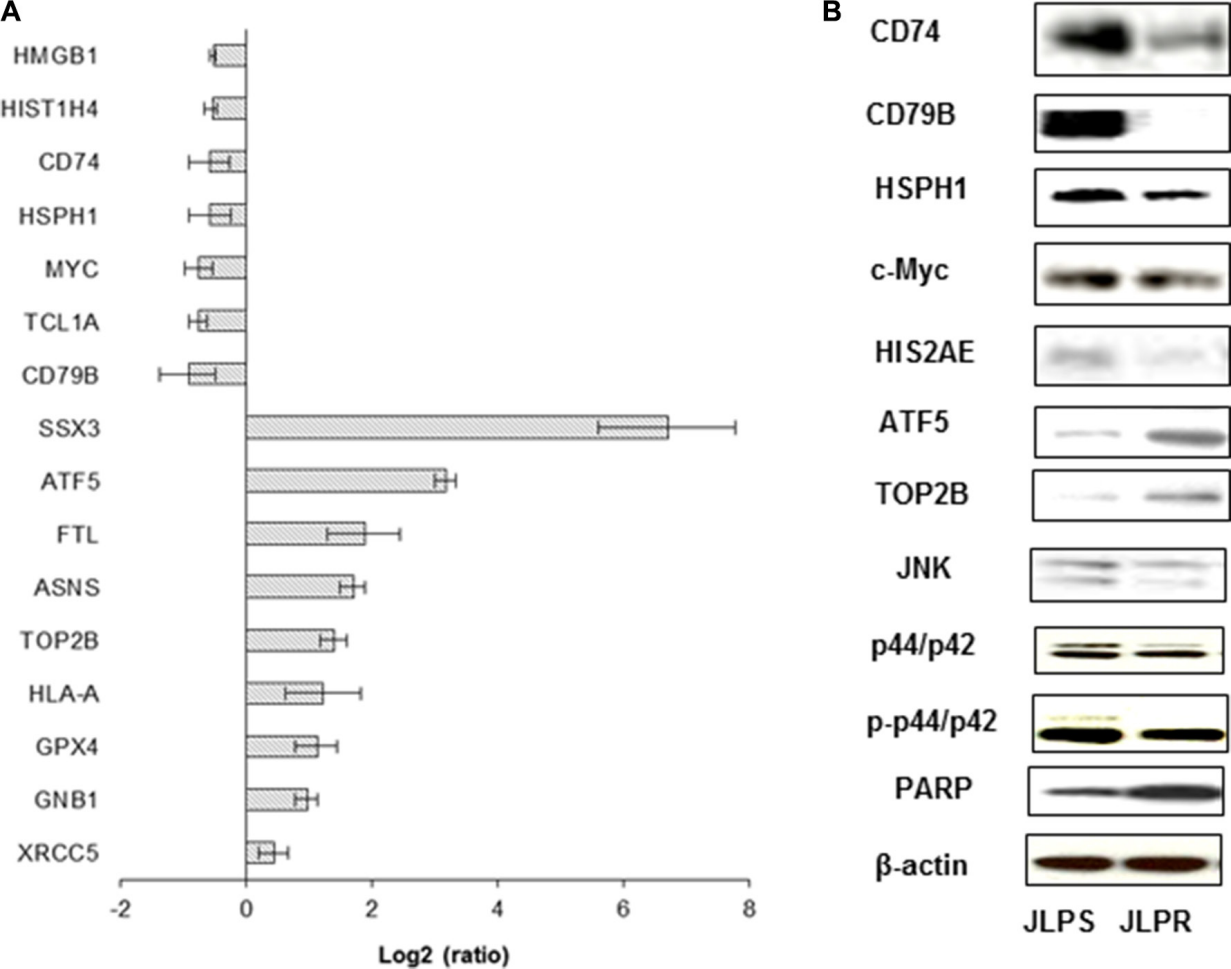
Real-time PCR and western blot analyses of differentially expressed genes and protein expression levels of JLPS and JLPR cells treated with ascorbate (**A**) Real-time PCR analysis of HMGB1, HIST1H4, CD74, HSPH1, c-Myc, TCL1A, CD79B, SSX3, ATF5, ASNS, FTL, TOP2B, HLA-A, XRCC5, GNB1, ATP5, and GPX4. Results were normalized using 18S ribosomal RNA. (**B**) Western blot analysis of CD74, CD79B, ATF5, TOP2B, HSPH1, HIS2AE, JNK, p44/p42, p-p44/p42, PARP, and c-Myc expression in JLPS and JLPR cells. β-actin was used as the loading control.

qRT-PCR revealed that the antioxidant genes *CAT* and *GPX4* (Figure 2E), the DNA repair genes *XRCC5* and *TOP2B*, and asparagine synthetase (*ASNS*), *GNB1*, *SSX3*, *ATF5*, *HLA-A*, and *FTL* were upregulated in JLPR cells (Figure 4A). Moreover, qRT-PCR and western blot analyses revealed that the expressions of *HMGB1*, *c-Myc*, *HIST1H4*, *CD74*, *CD79B*, and *HSPH1* were suppressed in JLPR cells (Figure 4A and Figure 4B). The functional activity assay showed that the activity of *CAT* and *GPX* increased in resistant JLPR cells adapted to up to 1 mM ascorbate (Figure 2G and Figure 2H).

## DISCUSSION

In the present study, we demonstrated that resistance to ascorbate reduced drug accumulation, decreased apoptosis, and increased glucose transport, anti-oxidation activity, and DNA repair. Compared with JLPS cells, JLPR cells generated smaller amounts of ROS than JLPS cells with ascorbate treatment (Figure 3C). Produced intracellularly as a byproduct of oxidative metabolism, under normal conditions H_2_O_2_ is reduced to water by *CAT*, *GPX*s, and peroxiredoxins [23]. In contrast, JLPR cells had a wide array of enzymatic and non-enzymatic antioxidants, including *GPX*, *CAT*, and glutathione. Other studies have revealed that *CAT* activity increases in H_2_O_2_-resistant fibroblasts and that cell death is prevented by cellular or extracellular *CAT* [24, 25]. Overexpression of the corresponding genes may dramatically increase cells' resistance to the ascorbate-induced oxidative damage of lipids, proteins, and DNA.

In our study, we found that JLPR cells were resistant to H_2_O_2_ and that the effects elicited by ascorbate or H_2_O_2_ in these cells were strikingly similar. Moreover, ascorbate and H_2_O_2_ exposed to JLPR cells resulted in reduced cell cycle arrest in G2/M phase, but increased glucose uptake compared with that in JLPS cells. We also found that Glut1 and Glut3 were primarily responsible for glucose transport in JLPR cells and that the amounts of these transporters were higher in JLPR than JLPS cells. Smith et al. found that increased glucose transport was associated with fluorouracil resistance in MCF7 cells and that *Glut*1 was increased in fluorouracil-resistant cells [26]. These results imply that JLPR cells overcame the role of H_2_O_2_ in mediating glucose uptake activation by activation of the expression of glucose transporters. We also investigated whether ascorbate treatment of JLPR and JLPS cells resulted in different patterns of gene regulation with oxidative stress pathways induced by reactive oxygen species. After treatment with ascorbate, the expression of c-Myc was downregulated in JLPR cells but overexpressed in JLPS cells (Figure 4D). c-Myc activation can induce sufficient DNA damage to elicit a stress response and induce the accumulation of cells with a 4N DNA content [27], which is consistent with our finding that JLPS cells treated with ascorbate induced sufficient DNA damage to cause cell cycle arrest in the G_2_/M phase. Following treatment with ascorbate, cleaved *PARP* was present in JLPS cells and native *PARP* was overexpressed in JLPR cells. We further analyzed the functional networks and gene pathways involved in ascorbate resistance. Although only five genes (*CD79B, ATF5*, *Myosin*, *MHC*, and *SAP18*) with similar and overlapping signature patterns were represented in the signaling pathways (phosphoinositide kinase signaling, *Cdc42* signaling, DNA methylation and transcriptional repression signaling, polyamine regulation signaling, and integrin-linked kinase signaling), each of their gene products are functionally involved in controlling cell growth and cell death and have been suggested to play roles in ascorbate resistance in JLPR cells. Real-time PCR and immunoblot analysis confirmed the expression of *ATF5* in JLPR cells. ATF5, which is part of the *ATF/cAMP*-response element-binding protein gene family, may play a role in protecting cells from amino acid limitation or arsenite-induced oxidative stress. Previous studies have shown that *ATF5* loss-of-function induces glioma cell death in culture and *in vivo* [28, 29]. Taken together, these results suggested that *ATF5* induces ascorbate resistance in lymphoma cells by activating transcription factors.

In conclusion, we found that in parental JLPS cells—but not in JLPR cells—treatment with pharmacologic ascorbate or H_2_O_2_ induced cell cycle arrest in the G_2_/M phase and *PARP* cleavage, decreased glucose uptake, and activated *c-Myc* pathway. In addition, the gene expression profiles of JLPS cells and JLPR cells were different. The differentially expressed genes in microarrays, which mediated multiple cytotoxic pro-oxidant stress pathways, were also verified at the levels of mRNA, protein, and functional activity by real-time PCR, western blotting, and enzyme activity analyses, respectively. These target genes from vitamin C–induced resistant cells may serve as novel biomarkers for identifying cancer patients who may benefit from pharmacologic ascorbate treatment.

## MATERIALS AND METHODS

### JLPS and JLPR Burkitt lymphoma cells

The Burkitt lymphoma cell line JLPS and ascorbate-resistant JLPR cells were maintained in RPMI 1640 medium (Mediatech, Inc., Herndon, VA, USA) with 10% inactivated FBS, 2 mM L-glutamine, and 50 μM β-mercaptoethanol. JLPR cells were maintained with 1 mM ascorbate. To develop cells resistant to ascorbate, we incubated JLPS cells with ascorbate in a stepwise manner, gradually increased from 100 μM to 1 mM over 6 months.

### O_2_ electrode assay of H_2_O_2_ concentration

The amount of H_2_O_2_ in the culture medium was measured using an O_2_ electrode (Hansatech Instruments, Ltd., Norfolk, UK) as described previously (24). Tested samples of cell culture medium that had been treated with 1 mM ascorbate or H_2_O_2_ for various times were placed in the reaction cells. After equilibration of the samples, 100 μl of catalase (CAT) solution (10,000 U/ml) was microinjected into the cells. Calibration curves were obtained by measuring the amount of O_2_ released in the medium.

### Assessment of glucose transport

After JLPS and JLPR cells (2 × 10^6^) were treated with ascorbate or H_2_O_2_, the cells were washed with PBS and incubated with 250 nM 2-deoxy-D-[1,2-^3^H] glucose (2-DG) (Sigma, St. Louis, MO, USA). The cells were washed with ice-cold PBS containing phloretin (100 μM), and dissolved in 300 μl of 0.1 N NaOH, 1% CHAPS. 2-DG uptake was measured using a scintillation counter.

### Measurement of intracellular ascorbate, CAT, and glutathione peroxidase (GPX)

After JLPS and JLPR cells were incubated with ascorbate, the cells were washed with PBS and harvested. The cell pellets were resuspended in 60% methanol and centrifuged at 15,300 rpm for 20 min. A HPLC system was used to assess the amount of intracellular ascorbate. For CAT activity, JLPS and JLPR cells (1.5 × 10^6^ cells) were centrifuged at 1,000 *g* for 10 min at 4°C. The cell pellet was sonicated in 300 μl of cold buffer comprising 50 mM potassium phosphate and 1 mM ethylenediaminetetraacetic acid and centrifuged at 10,000 *g* for 15 min. CAT activity in 20 μl of the resulting supernatant was assessed using a CAT assay kit (Calbiochem, San Diego, CA, USA) according to the manufacturer's instructions. Intracellular GPX4 activity was measured using a cellular GPX assay kit (Calbiochem) according to the manufacturer's instructions.

### Transcriptomic profiles

After quality assessment using the Agilent NanoChip Bioanalyzer assay, total RNA from cell lines was labeled using Agilent's Low RNA Input Labeling Kit, which involves reverse transcribing the mRNA to produce cDNA and then transcribing in the presence of Cy3-CTP or Cy5-CTP to produce labeled cRNA. Labeled cell line cRNA was paired with the differentially labeled normal colonic epithelial cRNA and, using the Agilent *In situ* Hybridization Kit, hybridized to the Agilent 44K Whole Genome Oligo Microarray (G4112A) for 17 hours at 60°C according to the manufacturer's protocol. The arrays washed in 2X SSC, 0.005% Triton × 102 for 10 minutes, 0.006 × SSPE, 0.005% N-lauroylsarcosine and coated with Agilent's Stabilization and Drying Solution. Arrays were scanned on Agilent DNA Microarray scanner.

### Pathway analysis

To determine the potential specific pathways based on changes in gene expression, we used the Ingenuity Pathway Analysis (IPA) software program (Ingenuity, Redwood City, CA) as described previously [21, 30].

## SUPPLEMENTARY MATERIALS TABLES




