# Supplementary material for: Transcriptomic and functional pathways analysis of ascorbate-induced cytotoxicity and resistance of Burkitt lymphoma
Source: Oncotarget. 2016 Aug 31;7(39):63950–9. doi: 10.18632/oncotarget.11740 (PMC5325416; doi:10.18632/oncotarget.11740)
Supplement: Supplementary file 1 [file oncotarget-07-63950-s001.pdf]

Transcriptomic and functional pathways analysis of ascorbate-induced cytotoxicity and resistance of Burkitt lymphoma

Supplementary Materials

MATERIALS AND METHODS

Cytotoxicity assay

The 3-(4, 5 dimethylthiazol-2-yl)-2,5 tetrazolium bromide (MTT) assay was used to assess cytotoxicity. JLPS and JLPR cells were plated in 96-well plates at 10^4 cells/well and grown overnight in a 5% CO_2 incubator at 37°C. Next, the cells were treated with various concentrations of ascorbate or H_2O_2 for 1 h. The cells were then collected, washed with PBS, and incubated in RPMI 1640 culture medium for an additional 24 h. The cells were then washed again with PBS three times and incubated in RPMI 1640 medium, then 10 µl of MTT (5 mg/ml) was added. After 4 h, 100 µl of DMSO was added to each well and measured at 570 nm.

Cell cycle analysis

Flow cytometry was used to determine cell cycle distribution. JLPS and JLPR cells (2.5×10^5/ml) were treated with ascorbate or H_2O_2 for 1 h. Then, the cells were washed with PBS, fixed in 95% ethanol, washed with 1% BSA in PBS, and resuspended in 1.0 g/ml of RNase. The cells were then stained with 50 µg/ml propidium iodide and analyzed using a FACSCalibur flow cytometer (BD Biosciences, San Jose, CA, USA). Flow cytometry data were analyzed using the ModFit DNA analysis software program (Verity Software House, USA).

RNA purification

RNA was puried from the JLPS and JLPR cells using Trizol LS reagent (Invitrogen, Carlsbad, CA, USA) and the RNeasy mini kit (Qiagen, Valencia, CA, USA). The quality of the purified RNA was assessed using an Agilent 2100 bioanalyzer (Agilent Technologies, Waldbronn, DE, USA).

Quantitative real-time polymerase chain reaction analysis

Total RNA (1 µg) was reverse transcribed to cDNA using the First Strand cDNA synthesis kit (Invitrogen). Quantitative real-time polymerase chain reaction (qRT-PCR) was performed using the ABI Prism 7900 HT sequence detection system and the Taqman Universal PCR master mix (both from Applied Biosystems, Foster City, CA, USA). qPCR results were analyzed using the Sequence Detector software program version 2.0 (Applied Biosystems, Grand Island, NY) and normalized using 18S ribosomal RNA.

Immunoblots

Cell lysates buffer were added to the cells, which were immunoblotted with antibodies against *PARP*, cleaved *PARP*, *HIS2AE*, *c-Myc*, *p44/ p42*, phosphorylated *p44/42*, *JNK*, *OCT1* (all from Cell Signaling Technology, Danvers, MA), *CAT*, *HSP105*, *CD74*, *CD79B*, and *β-actin* (all from Santa Cruz Biotechnology, Santa Cruz, CA, USA). Nuclear protein was extracted using the NE-PER nuclear and cytoplasmic extraction kit (Thermo Fisher Inc., Waltham, MA, USA) according to the manufacturer's instructions.

Microarray data analysis

The microarray images were analyzed using the GenePix software program version 5.1 (Molecular Devices, Sunnyvale, CA). The resultant gene lists and associated expression values were loaded into the NCI Microarray Facility mAdb database. We calculated the mean log_2-transformed ratio of genes expressed in JLPR cells to that in JLPS cells for the results from experiments performed in triplicate. The mean values were calculated by taking the antilog as the ratio of the gene expression measures of resistant cells to those of their parental cells.

Statistical analysis

Statistical values are presented as means ± standard deviations. The Student *t*-test was used to assess differences between groups. Results were considered significant at $P < 0.05$.

Supplementary Table S1: Gene expression profiles in JLPR cells. See Supplementary_Table_S1
