# Supplementary material for: Transcriptomic and functional pathways analysis of ascorbate-induced cytotoxicity and resistance of Burkitt lymphoma
Source: Oncotarget. 2016 Aug 31;7(39):63950–9. doi: 10.18632/oncotarget.11740 (PMC5325416; doi:10.18632/oncotarget.11740)
Supplement: Supplementary file 2 [file oncotarget-07-63950-s002.docx]

1. **Supplementary Table S1.** Gene expression profiles in JLPR cells.

| **Gene** | **Fold change*** | **UniGene** | **Description** |
| --- | --- | --- | --- |
| *SSX3* | 4.41 | Hs.558445 | Synovial sarcoma, X breakpoint 3 (SSX3), transcript variant 1 |
| ATF5 | 4.41 | Hs.9754 | activating transcription factor 5 (ATF5), mRNA. |
| FTL | 2.87 | Hs.433670 | ferritin, light polypeptide (FTL), mRNA. |
| SSX9 | 2.54 | Hs.553667 | synovial sarcoma, X breakpoint 9 (SSX9), mRNA. |
| DJ681N20.2 | 2.48 |  |  |
| HLA-A | 2.47 | Hs.181244 | major histocompatibility complex, class I, A (HLA-A), mRNA. |
| SSX6 | 2.39 | Hs.511998 | synovial sarcoma, X breakpoint 6 (SSX6), mRNA. |
| ASNS | 2.37 | Hs.489207 | asparagine synthetase (ASNS), transcript variant 2, mRNA. |
| SSX1 | 2.26 | Hs.434142 | synovial sarcoma, X breakpoint 1 (SSX1), mRNA. |
| TOP2B | 2.25 | Hs.475733 | topoisomerase (DNA) II beta 180kDa (TOP2B), mRNA. |
| HPS4 | 2.24 | Hs.474436 | Hermansky-Pudlak syndrome 4 (HPS4), transcript variant 2, mRNA. |
| HLA-A | 2.22 | Hs.181244 | Major histocompatibility complex, class I, A |
| HPS4 | 2.20 | Hs.474436 | Hermansky-Pudlak syndrome 4 (HPS4), transcript variant 2, mRNA. |
| SSX4 | 2.19 | Hs.558402 | synovial sarcoma, X breakpoint 4 (SSX4), transcript variant 2, mRNA. |
| HLA-A | 2.19 | Hs.181244 | major histocompatibility complex, class I, A (HLA-A), mRNA. |
| LOC649853 | 2.17 |  | PREDICTED: similar to HLA class I histocompatibility antigen |
| TKT | 2.17 | Hs.89643 | transketolase (Wernicke-Korsakoff syndrome) (TKT), mRNA. |
| TMEM45A | 2.16 | Hs.126598 | Transmembrane protein 45A |
| IGLL1 | 2.13 | Hs.348935 | immunoglobulin lambda-like polypeptide 1 (IGLL1), transcript variant 2, |
| SSX2 | 2.11 | Hs.558712 | synovial sarcoma, X breakpoint 2 (SSX2), transcript variant 2 |
| C5orf13 | 2.11 | Hs.36053 | chromosome 5 open reading frame 13 (C5orf13), mRNA. |
| SSX4 | 2.04 | Hs.558402 | synovial sarcoma, X breakpoint 4 (SSX4), transcript variant 2 |
| VDAC1 | 1.99 | Hs.519320 | voltage-dependent anion channel 1 (VDAC1), mRNA. |
| HLA-A | 1.97 | Hs.181244 | major histocompatibility complex, class I, A (HLA-A), mRNA. |
| CCDC69 | 1.96 | Hs.412117 | coiled-coil domain containing 69 (CCDC69), mRNA. |
| ZFP57 | 1.92 | Hs.156326 | PREDICTED: zinc finger protein 57 homolog (mouse) (ZFP57), |
| CXorf6 | 1.92 | Hs.20136 | Chromosome X open reading frame 6 |
| MYC | -1.77 | Hs.202453 | v-myc myelocytomatosis viral oncogene homolog (avian) (MYC),mRNA |
| HIST1H2AE | -1.89 | Hs.121017 | histone cluster 1, H2ae (HIST1H2AE), mRNA. |
| HIST1H2BK | -1.93 | Hs.437275 | histone cluster 1, H2bk (HIST1H2BK), mRNA. |
| POMP | -1.96 | Hs.268742 | proteasome maturation protein (POMP), mRNA. |
| MYL6 | -1.96 | Hs.632717 | myosin, light chain 6, alkali, smooth muscle and non-muscle (MYL6) |
| RPS7 | -1.97 | Hs.546287 | ribosomal protein S7 (RPS7), mRNA. |
| H2BFS | -1.97 | Hs.473961 | H2B histone family, member S |
| SNORD35A | -1.97 |  | small nucleolar RNA, C/D box 35A (SNORD35A) on chromosome 19. |
| RGC32 | -2.09 | Hs.507866 | response gene to complement 32 (RGC32), mRNA. |
| LOC432369 | -2.11 |  | ATP synthase, H+ transporting, mitochondrial F1 complex, |
| SAP18 | -2.13 | Hs.524899 | Sin3A-associated protein, 18kDa (SAP18), mRNA. |
| LOC646192 | -2.22 |  | PREDICTED: similar to nucleolar protein 5A (LOC646192) |
| ALOX5AP | -2.28 | Hs.507658 | arachidonate 5-lipoxygenase-activating protein (ALOX5AP) |
| CD79B | -2.41 | Hs.89575 | CD79b molecule, immunoglobulin-associated beta (CD79B), |
| LOC391158 | -2.55 | Hs.632494 | PREDICTED: similar to Transcription factor Dp-1 |
| CD79B | -2.62 | Hs.89575 | CD79b molecule, immunoglobulin-associated beta (CD79B) |
| HMGB1 | -2.68 | Hs.434102 | high-mobility group box 1 (HMGB1), mRNA. |
| TCL1A | -3.17 | Hs.2484 | T-cell leukemia/lymphoma 1A (TCL1A), mRNA. |
| HSPH1 | -3.23 | Hs.36927 | heat shock 105kDa/110kDa protein 1 (HSPH1), mRNA. |

*Fold change refers to the expression level of JLPR cells compared with parental JLPS cells
